# Report of Surgical Cases at the Buffalo General Hospital

**Published:** 1862-08

**Authors:** J. R. Lothrop

**Affiliations:** Attending Surgeon


BUFFALO

### Medical and Surgical Journal

VOL. II. AUGUST, 1862. NO. 1.

### ORIGINAL COMMUNICATIONS.

ART. I.—*Report of Surgical cases at the Buffalo General Hospital. By* J. R. Lothrop, *Attending Surgeon.*

The following report of cases which were under treatment in the surgical wards of the Buffalo General Hospital, covers a period of four months, viz: from March 1st to July 1st. It is presented with comments on the cases, not with the expectation of presenting any very new experience, but with the purpose of setting forth the ordinary clinical facts which came under notice, having perhaps less variety and less novelty than might be met with at other periods of hospital service. Our hospitals afford the most favorable field for clinical observation, and the facts there met with, even if they do not go beyond the limits of the most ordinary experience, should be collected in some accessible form.

In the following list, the affections, local, or nearly so in their nature, are first mentioned, and secondly those more general. The different varieties which occur in a class will be spoken of in the remarks which follow:

| | | | |
|---|---|---|---|
| Wounds of the Scalp, | 2 | Affections of the Eye, | 8 |
| Affections of the Uterus, | 2 | Cystitis, | 1 |
| Disease of Prostate, | 1 | Orchitis, | 2 |
| Gonorrhœa, | 3 | Spematorrhœa, | 1 |
| Fissure of the Anus, | 1 | Hernia, | 2 |
| Fractures, | 5 | Ulcers, | 5 |
| Tumor benign, | 1 | | |

Affections more general or constitutional.

| | | | |
|---|---|---|---|
| Disease of the bones and joints, | 5 | Paralysis, - - - | 4 |
| Erysipelas, - - - | 3 | Syphilis, - - - - | 11 |
| Tumors malignant, - - | 2 | | |

1.—The wounds of the scalp were not severe. In one sutures were employed, the wound readily uniting without accident. The use of sutures in such cases is not commonly recommended, for the reason that they are liable to excite erysipelas of the scalp. As far as the writer has had any experience, no such result has followed their use. We may mention the statement of a surgeon of large experience, that he had always practiced stitching of the scalp and had never seen it followed by erysipelas.

2.—The affections of the eye included three cases of what I may term chronic ophthalmia, three cases of cataract, one of inflammation of the cornea, one of injury.

The three cases of chronic ophthalmia were the result of purulent conjunctivitis, in which in severe cases the disease is not limited to the conjunctiva, but other structures become implicated, especially the iris and cornea; the former becoming adherent to the cornea, thereby fixing the pupil, and the latter being rendered opaque as a result of ulceration or perforation. In two of these cases these results had followed, and vision had therefore been seriously impaired. In the third the opacity was confined to the upper half of the cornea, being caused by the roughness of the upper lid. These cases offer but little hope to treatment, but it is to be borne in mind that surprising improvement will often take place, under judicious management. We are not to despair of improvement in cases of opacity of the cornea to a considerable extent, if we allow time for changes which are always slow, and are not forced into violent methods. Especially is the opacity caused by rough lids benefitted by treatment which cures the same. For this solid sulphate of copper applied lightly once daily, is to be preferred. Mild astringents—mineral, not vegetable, for the latter seldom benefit—are only to be used. In the above cases mild solutions of zinc and borax witn occasional use of a solution of atropine were employed.

In the cases of cataract two were of the hard variety, occurring generally in men past middle age. In these no operation was deemed advisable, as vision was tolerably good, though both eyes were affected. In the third case the operation for depression had been made by my predecessor, but in a short time was followed by a greater opacity than had previously existed, forming what is spoken of as secondary capsular cataract; in fact the only variety of true capsular cataract, the capsule becoming opaque and obstructing the pupil. As this opaque capsule in general is sooner or later absorbed, it was deemed advisable to wait awhile. After a period of several weeks no improvement had taken place, but at that time the patient left the hospital. In this case, however, and in all such cases, an operation for the removal of the opaque capsule from the pupil would have been proper, after allowing sufficient time to ascertain the probability of absorption.

The single case of coneitis occurred in a scrofulous boy, and consisted of several minute ulcerations on the cornea, attended with great intolerance of light, and some affection of the iris. As is common it was not a simple local affection, being partly constitutional in its origin. It was treated with mild astringents, with a solution of atropine to ease pain and remedy intolerance of light, and iodide of potash internally. It should be borne in mind that the constitutional is quite as important as the local treatment in these cases. The progress was slow, but in a few weeks there was more tolerance of light, and less injection of the sclerotica around the cornea. The small opacities resulting from ulceration of the cornea would not be likely to disappear except after a considerable time. The cornea does not possess a high order of organization, and reparative changes are therefore slow, hence we should always be prepared to find the progress in coneitis somewhat tardy. The boy is still under treatment.

One case of traumatic affection of the eye occurred. In this ammonia was the injurious agent. The patient stated that a bottle containing strong aqua ammoniæ was held near the face, and the cork being suddenly blown out the vapor was brought in contact with the face. Both eyes were injured, but only one seriously. The left eye was highly inflamed, and partial adhesion of the upper lid to the eyeball had taken place. A lotion of tepid water was applied to the eye and the patient kept in bed. But he desiring more activity only remained two days, so that the result cannot be stated. The result to be expected is adhesion of the lids to the eyeballs. It is unfortunate that very little can be done in the way of prevention, or to obviate it when it has taken place. Although the adhesion may be divided, yet it is almost impossible to prevent re-union.

3.—Of the two cases of disease of the uterus one was schirrus of the cervix, and one ulceration of the cervix, with enlargement and induration of the whole organ. The former upon examination exhibited the usual narrowed, hardened, almost cartilagenous condition of the vagina; an irregular, hardened cervix, with a large os. This patient was about forty years of age, and mother of several children. She suffered from very severe pain in the region of the uterus, besides sympathetic affection of the bladder and rectum, and an offensive discharge. The only treatment adopted was the giving of opiates, of which she took large quantities, to ease pain, and an injection of dilute chlorinated soda to correct the unpleasant odor. She left the hospital after remaining a few months. In the latter the ulceration was an inch in diameter; there was abundant muco-purulent discharge and occasional bleeding, with dull, heavy pain. After treatment for considerable time, by the use of iodine in form of injection and internally, varied with the use of tonics and the application of the solid nitrate of silver twice a week, the patient recovered and left the hospital.

4.—The case of cystitis occurred in a young man about twenty years of age. It was of several years' duration, but he had no definite idea of the cause. The symptoms were almost entire incontinence of urine, with occasional hæmorrhage and a constant ropy mucous secretion. The passage of an instrument caused great pain, as did also the injection of a small quantity of liquid. The capacity of the bladder was greatly diminished, and the coats probably thickened. The neck of the bladder seemed thickened, as the catheter met with a momentary resistance, and seemed to slip through a hard ring on its entrance. From the bleeding it was conjectured that the mucous coat of the bladder must have points of ulceration but it might have arisen from the congested and thickened mucous coat.

In this case the treatment was mainly local, and for a time seemed beneficial. The bleeding diminished and the tolerance of the bladder became greater, so that it would allow of a moderate accumulation of urine. The appearance of the patient too, changed greatly for the better, the countenance losing the expression of pain and anxiety which it wore when he first entered the hospital. The injections employed consisted first of ten drops of muriatic acid in an ounce of water. During the use of which the urine lost its alkaline reaction, and the amount of mucus was greatly diminished. Afterwards tannin, ten grains to the ounce of water was used, and that followed by injections of nitrate of silver ten grains to the ounce, which was continued for about a month. More benefit was apparent from the nitrate of silver than from the other substance. The injections were made at first every other day, and afterwards twice a week. During the treatment the benefit was decided enough to warrant perseverance. The time during which treatment was continued was about two months.

5.—The case enumerated as disease of the prostate gland was supposed to be abscess of the gland, though the acute period of the affection had passed when the patient—a man forty-five years of age—was admitted. His description of what took place before he came in was, pain, throbbing deep in the perenium, followed by swelling, a good deal of fever and chills, with pain in defecation, and some obstruction to the flow of urine, as well as pain in its passage. The urine moreover let fall after a time an abundant whitish deposit. The cause was attributable to cold from wetting the feet. When admitted there was considerable induration and swelling in the perineum, the pain during defecation had not entirely disappeared, and the urine contained a puriform deposit in small quantity. The patient was much reduced by pain and confinement in bed. This was thought to have been a case of abscess in the prostate, which had discharged itself into the urethra. While the induration continued in the perineum the patient was kept in bed and a poultice applied. Tonics were given to repair his strength, which was impaired, this being then the only indication. The particular remedies given were the muriated tincture of iron, in combination with the chlorate of potassa. After a short time he was restored to his usual health.

6.—Three cases of gonorrhœa, two of which were complicated with swelling of the testicle. In these cases cubebs in powder a scruple three times daily, and mild injections daily were employed; nitrate of silver one or two grains to the ounce in the early, and sulphate of zinc three or four grains to the ounce in the latter stage. The stronger injections often recommended were not deemed advisable. The patient was directed to urinate just before injection was made, for the double purpose of removing the purulent secretion from the urethra, and thereby aiding the more thorough application of the solution injected to the mucous lining of the urethra, and secondly to prevent the washing away of the solution by the passage of urine soon after the injection had been made. This point was deemed worthy of attention in the treatment in all cases. The orchitis which followed, after a few days' treatment, at first by cold application, aided by rest in bed, and afterwards by the application of straps to the testicle, speedily disappeared. The swelling of the testicle following gonorrhœa usually disappears in a short time aided by rest or suspension, but after the acute symptoms are past, strapping very speedily reduces its bulk.

7.—In connection with this, the case of spermatorrhœa may be mentioned. This was a case in which an occasional involuntary seminal emission at night, had so wrought upon the morbid sensibility of a young man as to seriously affect his health and spirits. The treatment of such cases is more largely moral than physical. Benefit has been observed in such cases from the use of lupulin and strychnine, and these were given, but the influence is so essentially moral, that we shall effect more by trying to divert the mind from morbid excitement, and by endeavoring to convince of the comparatively harmless nature of the affection than by the adminis·tration of medicine. The patient left in a short time without being greatly benefitted by advice or remedies.

8.—The case of fissure of the anus was simply an unhealed incision after the operation for the cure of fistula in ano. The operation had bcen done, by Dr. Eastman, about six months previously. The fissure healed readily when its sides were kept from contact by being packed with lint, but the patient had not fully recovered the power of retaining the contents of the rectum. At the present time, nearly nine months, control over the sphincter ani is not fully restored.

9.—The two cases of hernia were both oblique inguinal hernia, of long standing. The first was of moderate size, easily reduced, and giving little trouble except on unusual exertion. This patient was provided with a truss by a contribution of several charitable ladies, and was thus enabled to engage in an active occupation. The second was strangulated, and became the subject of an operation. The history of the case is as follows: M. W., a German, coachman, aged twenty-nine, was admitted to the surgical ward of the hospital about 10 P. M. Soon after, I was called, and upon arrival found the case to be one of strangulated hernia, having as nearly as I could ascertain been irreducible for about seven or eight hours. The hernia being scrotal had existed about three years; had never given any trouble, being always reducible, and had never been confined by a truss. On the day of his admission, May 10th, in the morning, it was of its usual size, as large as a hen's egg. The patient was engaged in the latter part of the day at the stable with which he was connected, handling grain, and had made unusual exertion. Early in the afternoon experiencing some pain in the region of the tumor, his attention was called to it, when he found it incapable of reduction. He remained at the stable till six o'clock P. M., up to which time he suffered great pain and made violent efforts to reduce the tumor, until very much exhausted by the pain and his exertions at reduction. At the time mentioned, having failed to reduce the tumor, he walked to his house, a short distance, and immediately went to bed. He was so exhausted and irritable that it was some time before his wife ascertained what was the matter with him. After a while a physician was summoned, and Drs. Loomis and Brown came to his assistance. They failed to reduce the hernia after as long trial as they deemed judicious, and advised that he be removed to the hospital. When first seen by me the tumor had attained a most enormous size, nearly as large as a child's head, was tense and tympanitic and very red, as I supposed from handling. The patient was restless, showing evident signs of prostration or shock; skin cool and moist, and pulse feeble. He had vomited several times, and had had also a passage from the bowels below. An injection of warm water was ordered which, having no influence, after a brief trial of taxis, it seemed best to relieve the strangulation by an operation, without farther delay. Chloroform was therefore administered by the house surgeon, Dr. Whitbeck, and when the patient was fully under its influence and complete relaxation produced, a last attempt at reduction was made, without success. The operation was accordingly commenced with the assistance of the house surgeon, the ward nurse and several friends of the patient. It being a large and old hernia it was thought best to make the incision as small as would suffice to accomplish the reduction. A small vertical incision an inch and a half or two inches in length was made just below the outer ring. Arriving at the peritoneum it was found thickened and the constriction was caused by it just outside of the ring, which appeared quite large and free. Several bands like threads were very tightly drawn across the tumor at this point, so that pouches sprang up between. An attempt was made to return the intestine without dividing the peritoneum, the finger being easily passed into the ring. This not proving successful the peritoneum was pinched up and carefully divided, a director passed beneath it, and the constricting bands divided. The finger was then passed into the opening and the intestine pushed inward. In a short time a gurgling and giving way were perceptible, and soon the whole intestine returned to the abdomen. The wound was closed by a few points of suture, a wet compress placed over it, and a bandage applied around the pelvis and thigh, forming a figure of eight. The patient was placed in bed and ordered one-half grain of morphine at once, to be repeated in three hours if he was restless.

The appearance of the intestine upon opening the peritoneum was that of intense congestion, it being almost claret colored. There was considerable fluid in the sac, and a slightly offensive odor.

At the visit on the following morning, 9 A. M., the nurse reported quiet and freedom from pain till 2 A. M. The patient then became restless and a dejection took place, which was bloody. His condition at that time was as follows: Pulse 120, rather small; skin cool, considerable thirst; abdomen full and rather sensitive to heavy pressure. This condition continued throughout the day, the patient taking a small amount of food. At night he was not perceptibly worse, fomentations having been continued to the abdomen throughout the day. During the night he became very restless, with continued moaning. The pulse became smaller and more frequent, the skin clammy and the abdomen more distended. He began to sink, and at the visit next morning was moribund, dying near noon following. On inspection at the morning visit, it was found that the incision had not united, and upon pressing the abdomen a yellow sero-purulent fluid flowed abundantly from it. No autopsy was made.

In review of this case these questions arise: Was an operation necessary? Could it have been delayed? What was the cause of death?

First, then, if the tumor was irreducible, it is plain that an operation was essential. The question of reducibility must be left to the judgment of the surgeon, though he may exaggerate the urgency. But if all reasonable means of reduction have failed, he must not wait in the hope that something favorable will turn up, rather than resort to the relief which is certain, though confessedly not without danger. Secondly, how long are we to wait before we decide that there is danger in delay? No definite time can be stated, and generally a small and recent hernia admits of least delay; old and large herniæ being less likely to give rise to urgent symptoms. But it is to be borne in mind that our course is to be determined rather by the symptoms, than by the character of the hernia, for in large as in small herniæ, symptoms of the most intense and dangerous character may set in, in a number of hours. In this case the symptoms were intense, and farther delay seemed only making the patient's chances less. Moreover, prolonged taxis, especially if violent, is likely to result in injury to the imprisoned intestine.

Thirdly, the death of the patient could hardly have been from peritonitis, as thirty-six hours, though not an impossibility, is yet rather a short interval for its fatal onset. In this case however some inflammation of the sac was to be looked for, as there is no probability that it was returned. In old herniæ the sac is not commonly reducible, having formed adhesions in the scrotum. The interior of the sac therefore was exposed to the irritant influence of the air. On the other hand, the dark color of the intestine, the time of incarceration, and the probable violence which the intestine had borne from the patient himself, the bloody discharge in consequence, and the time at which death occurred, favored the idea of death by gangrene of the intestine. The death by asthenia, without much pain, and especially without vomiting, resembled more the prostrating influence arising from the destructive change of a vital organ. Assuming what seems probable, there appears to be no good ground for supposing that delay would have contributed in any degree to a more favorable result; rather it seems to warrant the inference that a somewhat earlier resort to an operation would have improved the patient's chances. Though seven hours is not a long time, yet dangerous symptoms when the incarceration is complete, may arise in even less time.

[TO BE CONTINUED.]

